# Supplementary material for: Strategies for improving the performance of prediction models for response to immune checkpoint blockade therapy in cancer
Source: BMC Res Notes. 2024 Apr 9;17:102. doi: 10.1186/s13104-024-06760-5 (PMC11005243; doi:10.1186/s13104-024-06760-5)
Supplement: Supplementary file 1 — Additional file 1: Table S1. Feature selection with different algorithms. Figure S1. UMAP for the merged datasets based on (a) the original 18878 features and (b) the 135 features characterizing the pairwise relations of immune checkpoint genes. Table S2. Clinical Characteristics. Figure S2. Random forest - ROC plot for thecomparison of the result. Comparison of ROC curves with applying Random Forest on each single dataset. (A) Cross validation using single dataset Van Allen et al.; (B) Cross validation using single dataset Hugo et al.; (C) Cross validation using single dataset Riaz et al. Figure S3. Lasso - ROC plot for the comparison of the result. Comparison of ROC curves with applying Lasso on each single dataset. (A) Cross validation using single dataset Van Allen et al.; (B) Cross validation using single dataset Hugo et al.; (C) Cross validation using single dataset Riaz et al. Figure S4. XGBoost - ROC plot for the comparison of the result. Comparison of ROC curves with applying XGBoost on each single dataset. (A) Cross validation using single dataset Van Allen et al.; (B) Cross validation using single dataset Hugo et al.; (C) Cross validation using single dataset Riaz et al. Figure S5. ROC curves of the combined two datasets (Van Allen et al. and Riaz et al.) for all algorithms. [file 13104_2024_6760_MOESM1_ESM.pdf]

Appendices for "Strategies for Improving the Performance of Prediction Models for Response to Immune Checkpoint Blockade Therapy in Cancer"

Tiantian Zeng[1*], Jason Z. Zhang[2], Arnold Stromberg[1], Jin Chen[3], Chi Wang[4*]

[1]Department of Statistics, University of Kentucky, 725 Rose St, Lexington, 40536, KY, U.S..
[2] Wake Forest University, Winston-Salem, 27109, NC, U.S..
[3]Department of Medicine - Nephrology, University of Alabama at Birmingham, 703 19th St S, Birmingham, 35294, AL, U.S..
[4*]Department of Internal Medicine, University of Kentucky, 800 Rose St, Lexington, 40536, KY, U.S..

*Corresponding author(s). E-mail(s): zengtiantian009@gmail.com; chi.wang@uky.edu;
Contributing authors: zhanjz22@wfu.edu; stromberg@uky.edu; jichen@uabmc.edu;

Appendix A: List of feature selection and probability

Table S1: Feature selection with different algorithms

Index	Variable Name	Random Forest	Lasso	XGBoost
1	PD-1>PDL-1	0.18	1	0.21
2	PD-1>CTLA4	0.34	0.72	0.66
3	PD-1>CD28	0	0.44	0.34
4	PD-1>CD80	0.01	0	0.14
5	PD-1>CD86	0	0	0.04
6	PD-1>BTLA	0.02	0.35	0.12

Continued on next page

Table S 1 – continued from previous page

Index	Variable Name	Random Forest	Lasso	XGBoost
7	PD-1>CD200	0	0	0.01
8	PD-1>CD200R1	1	0.51	0.92
9	PD-1>CD27	0.02	0.93	0.05
10	PD-1>CD276	0	0.95	0
11	PD-1>CD40	0	0	0
12	PD-1>CEACAM1	0	0.03	0.02
13	PD-1>TIM-3	0	0.02	0
14	PD-1>IDO1	0	0.01	0.02
15	PD-1>IL2RB	0	0.02	0
16	PD-1>LAG3	0	0	0.03
17	PD-1>PD-1LG2	0	0	0.42
18	PD-1>PVR	0	0.07	0.05
19	PD-1>TIGIT	0	0.05	0.04
20	PD-1>HVEM	0	0.01	0.06
21	PD-1>TNFRSF18	0.98	0.01	0.76
22	PD-1>TNFRSF4	0.05	0.98	0.29
23	PD-1>TNFRSF9	0	0.01	0.22
24	PD-1>OX40L	0.81	0	0.64
25	PD-1>CD137L	0.83	0.44	0.55
26	PDL-1>CTLA4	0.32	0.16	0.24
27	PDL-1>CD28	0.02	0	0.23
28	PDL-1>CD80	0	0.06	0
29	PDL-1>CD86	0.01	0	0.19
30	PDL-1>BTLA	0	0	0
31	PDL-1>CD200	0.01	0	0.1
32	PDL-1>CD200R1	0	0.01	0.02
33	PDL-1>CD27	0.04	0.01	0.41
34	PDL-1>CD276	0	0.04	0
35	PDL-1>CD40	0	0.74	0
36	PDL-1>CEACAM1	0	0.13	0.02
37	PDL-1>TIM-3	0	0	0
38	PDL-1>IDO1	0.09	0	0.21
39	PDL-1>IL2RB	0	0	0.07
40	PDL-1>LAG3	0.28	0	0.25
41	PDL-1>PD-1LG2	0	0	0.22
42	PDL-1>PVR	0.11	0	0.41
43	PDL-1>TIGIT	0.1	0	0.5
44	PDL-1>HVEM	0	0.47	0
45	PDL-1>TNFRSF18	0	0.01	0.03
46	PDL-1>TNFRSF4	0.11	0	0.1
47	PDL-1>TNFRSF9	0	0	0

Continued on next page

Table S 1 – continued from previous page

Index	Variable Name	Random Forest	Lasso	XGBoost
48	PDL-1>OX40L	0.17	0	0.45
49	PDL-1>CD137L	0.93	0.07	0.64
50	CTLA4>CD28	0.03	0.76	0.3
51	CTLA4>CD80	0.89	0	0.64
52	CTLA4>CD86	0	0.3	0.07
53	CTLA4>BTLA	0	0	0.04
54	CTLA4>CD200	0.11	0	0.17
55	CTLA4>CD200R1	0.9	0.83	0.51
56	CTLA4>CD27	0.04	0.95	0.25
57	CTLA4>CD276	0	0	0
58	CTLA4>CD40	0	0	0.02
59	CTLA4>CEACAM1	0	0	0.02
60	CTLA4>TIM-3	0	0	0.04
61	CTLA4>IDO1	0.01	0.2	0.28
62	CTLA4>IL2RB	0	0.01	0.09
63	CTLA4>LAG3	0.98	0	0.87
64	CTLA4>PD-1LG2	0.03	0.96	0.43
65	CTLA4>PVR	0.14	0.02	0.24
66	CTLA4>TIGIT	0.1	0	0.21
67	CTLA4>HVEM	0	0.05	0
68	CTLA4>TNFRSF18	0.08	0.03	0.2
69	CTLA4>TNFRSF4	0.31	0.14	0.13
70	CTLA4>TNFRSF9	0.37	0.01	0.29
71	CTLA4>OX40L	0.22	0.99	0.44
72	CTLA4>CD137L	0.3	0.48	0.32
73	CD28>CD80	0	0	0.13
74	CD28>CD86	0.68	0.01	0.12
75	CD28>BTLA	0	0.68	0.01
76	CD28>CD200	0	0.08	0.03
77	CD28>CD200R1	0	0	0.06
78	CD28>CD27	0	0.01	0.24
79	CD28>CD276	0	0.04	0
80	CD28>CD40	0	0	0
81	CD28>CEACAM1	0	0.1	0.02
82	CD28>TIM-3	0.92	0.01	0.13
83	CD28>IDO1	0.01	0.99	0.15
84	CD28>IL2RB	0	0	0.09
85	CD28>LAG3	0.62	0.01	0.42
86	CD28>PD-1LG2	0.07	0.03	0.26
87	CD28>PVR	0	0.01	0.08
88	CD28>TIGIT	0.02	0	0.27

Continued on next page

Table S 1 – continued from previous page

Index	Variable Name	Random Forest	Lasso	XGBoost
89	CD28>HVEM	0	0	0
90	CD28>TNFRSF18	0	0	0.02
91	CD28>TNFRSF4	0.01	0	0.2
92	CD28>TNFRSF9	0	0.03	0.07
93	CD28>OX40L	0.04	0.03	0.19
94	CD28>CD137L	1	0	0.86
95	CD80>CD86	0	0.97	0
96	CD80>BTLA	0.03	0.21	0.17
97	CD80>CD200	0	0.01	0
98	CD80>CD200R1	0.55	0.04	0.63
99	CD80>CD27	0.01	0.48	0.12
100	CD80>CD276	0	0.25	0
101	CD80>CD40	0	0	0
102	CD80>CEACAM1	0.31	0	0.1
103	CD80>TIM-3	0	0.8	0
104	CD80>IDO1	0.03	0	0.11
105	CD80>IL2RB	0	0.04	0
106	CD80>LAG3	0.15	0.08	0.19
107	CD80>PD-1LG2	0.04	0	0.17
108	CD80>PVR	0	0.05	0.02
109	CD80>TIGIT	0	0.06	0.11
110	CD80>HVEM	0	0	0.08
111	CD80>TNFRSF18	0.05	0	0.17
112	CD80>TNFRSF4	0.22	0	0.36
113	CD80>TNFRSF9	0.01	0.03	0.16
114	CD80>OX40L	0.68	0.01	0.49
115	CD80>CD137L	0.81	0.18	0.66
116	CD86>BTLA	0	0.09	0
117	CD86>CD200	0	0.01	0.17
118	CD86>CD200R1	0	0.01	0
119	CD86>CD27	0	0	0.07
120	CD86>CD276	0	0.01	0
121	CD86>CD40	0.01	0	0.1
122	CD86>CEACAM1	0.02	0	0.34
123	CD86>TIM-3	0.01	0	0.06
124	CD86>IDO1	0.4	0.11	0.72
125	CD86>IL2RB	0.96	0.02	0.66
126	CD86>LAG3	0	0.61	0.04
127	CD86>PD-1LG2	0	0.01	0
128	CD86>PVR	0.28	0.07	0.69
129	CD86>TIGIT	0	0.45	0

Continued on next page

Table S 1 – continued from previous page

Index	Variable Name	Random Forest	Lasso	XGBoost
130	CD86>HVEM	0	0.02	0
131	CD86>TNFRSF18	0	0.88	0
132	CD86>TNFRSF4	0	0	0.01
133	CD86>TNFRSF9	0	0.04	0
134	CD86>OX40L	0	0	0
135	CD86>CD137L	0.01	0.01	0.03

Note: A total of 135 candidate features were considered, where each feature is the pairwise relation between two immune checkpoint genes. Numbers presented in the table are frequencies of features being selected by one of the three models (random forest, Lasso, or XGBoost) based on 10-fold cross validation as described in the method section.

Appendix B: UMAP plots

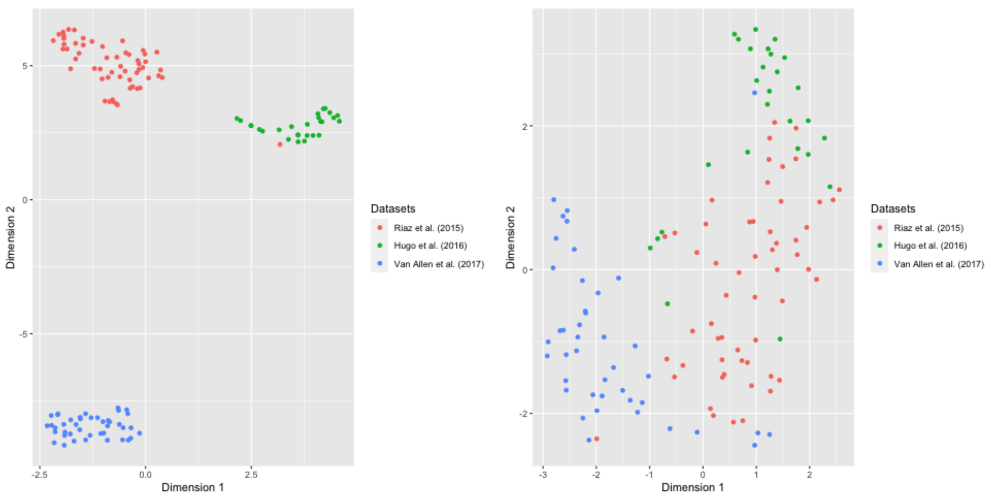

(a) UMAP for original datasets (b) UMAP for the transformed datasets

Figure S1: UMAP for the merged datasets based on (a) the original 18878 features and (b) the 135 features characterizing the pairwise relations of immune checkpoint genes.

Appendix C: Patient characteristics table

**Table S2 Clinical Characteristics**			
Characteristic	*Datasets*		
	Van Allen et al. (n=42)	*Hugo et al. (n=27)*	*Riaz et al. (n=56)*
Sex – no. (%)			
Female	14 (33.34)	8 (29.63)	N/A
Male	28 (66.67)	19 (70.37)	N/A
Age – median (range)	61 (22, 83)	62 (27, 84)	N/A
Metastasis – no. (%)			
M0	1 (2.38)	1 (3.70)	1 (1.79)
M1a	3 (7.14)	2 (7.41)	12 (21.43)
M1b	7 (16.67)	2 (7.41)	8 (14.29)
M1c	31 (73.81)	22 (81.48)	25 (44.64)
Unknown	0	0	10 (17.86)
Response			
non-response – no. (%)	28 (66.67)	21 (77.78)	45 (80.36)
response – no. (%)	14 (33.34)	6 (22.22)	11 (19.64)
Treatment history – no. (%)	N/A	N/A	Ipi-P: 30 (53.57)<br>Ipi-N: 26 (46.43)

Ipi-P refers to patients who had previously progressed on ipilimumab (Ipi) therapy, Ipi-N refers to patients who were Ipi-naïve.

Appendix D: ROC curves for models on each single dataset

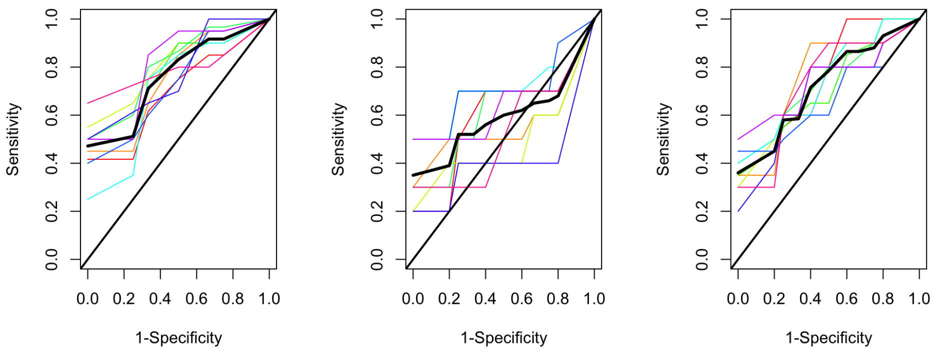

(a) Single dataset Van Allen et al.

(b) Single dataset Hugo et al.

(c) Single dataset Riaz et al.

Figure S2: **Random forest - ROC plot for the comparison of the result** Comparison of ROC curves with applying Random Forest on each single dataset. (A) Cross validation using single dataset Van Allen et al.; (B) Cross validation using single dataset Hugo et al.; (C) Cross validation using single dataset Riaz et al. Each colored dashed curve indicates one 10-fold cross validation replicate. The solid black curve indicates the average curve across ten replicates. Results were averaged across ten 10-fold cross-validations. The average area under the curve (AUC) were calculated over the ten replicates.

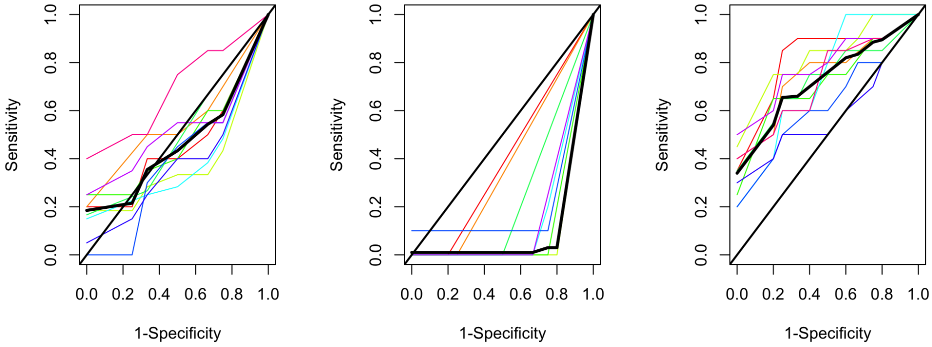

(a) Single dataset Van Allen et al.

(b) Single dataset Hugo et al.

(c) Single dataset Riaz et al.

Figure S3: **Lasso - ROC plot for the comparison of the result**
Comparison of ROC curves with applying Lasso on each single dataset.
(A) Cross validation using single dataset Van Allen et al.; (B) Cross validation using single dataset Hugo et al.; (C) Cross validation using single dataset Riaz et al.

Some of the Lasso ROC curves were not present, since some of the models did not select any RNA-seq pairs, with only intercept left and leading to an AUC of 0.5. Similarly, using XGBoost algorithm, some of the models did not select any feature, and thus non-tree model was detected. Hence, some ROC curves were not shown in Fig 4 (middle panel).

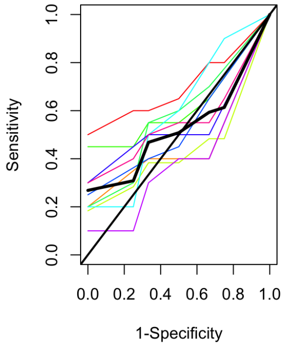

(a) Single dataset Van Allen et al.

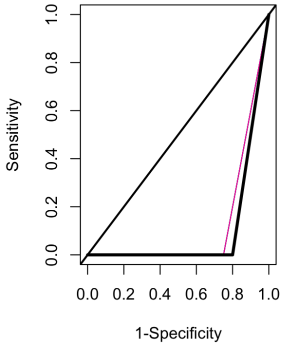

(b) Single dataset Hugo et al.

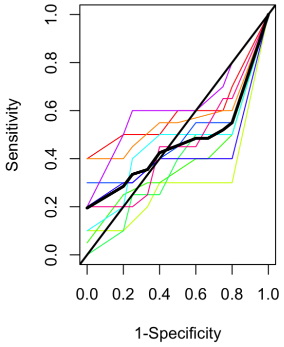

(c) Single dataset Riaz et al.

Figure S4: **XGBoost - ROC plot for the comparison of the result**
Comparison of ROC curves with applying XGBoost on each single dataset.
(A) Cross validation using single dataset Van Allen et al.; (B) Cross validation using single dataset Hugo et al.; (C) Cross validation using single dataset Riaz et al.

Appendix E: ROC curves for the models on combined datasets of Van Allen et al. and Riaz et al.

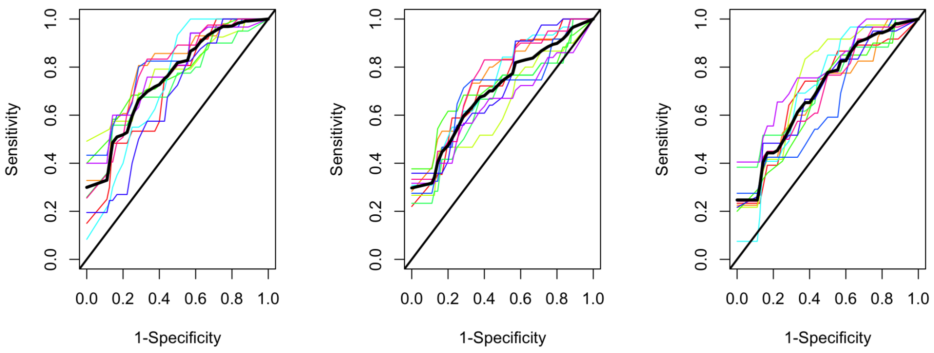

(a) ROC Curves for Random Forest

(b) ROC Curves for Lasso

(c) ROC Curves for XGBoost

Figure S5: **ROC curves of the combined two datasets (Van Allen et al. and Riaz et al.) for all algorithms.**

Each colored dashed curve indicates one 10-fold cross validation replicate. The solid black curve indicates the average curve across ten replicates. Results were averaged across ten 10-fold cross-validations. The AUC was calculated over the ten replicates.
